# The lead leads the way

**DOI:** 10.1007/s12471-021-01622-2

**Published:** 2021-08-27

**Authors:** S. Borges, J. I. Moreira

**Affiliations:** Cardiology Department, Centro Hospitalar de Trás os Montes e Alto Douro, Vila Real, Portugal

An 86-year-old diabetic and hypertensive female with permanent atrial fibrillation (AF) presented to the emergency department with recurrent pre-syncope and one episode of syncope. The baseline electrocardiogram showed AF with a ventricular rate of 47 beats/min. Holter monitoring confirmed AF with a slow ventricular rate. The patient was then referred for single-chamber pacemaker implantation but the procedure was not uneventful. Fig. 1 shows the final position of the lead at the end of the procedure. What is the most likely diagnosis?

**Fig. 1:**
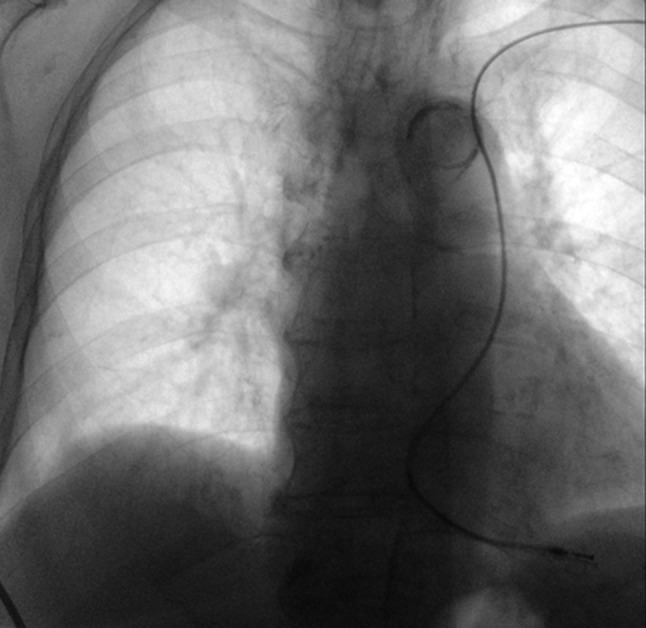
Position of the lead following single-chamber pacemaker implantation

## Answer

You will find the answer elsewhere in this issue.

